# The Regulation of Thermodynamic Behavior and Structure of Aluminosilicate Glasses via the Mixed Alkaline Earth Effect

**DOI:** 10.3390/ma18153450

**Published:** 2025-07-23

**Authors:** Lin Yuan, Xurong Teng, Ping Li, Ouyuan Zhang, Fangfang Zhao, Changyuan Tao, Renlong Liu

**Affiliations:** 1 College of Chemistry and Chemical Engineering, Chongqing University, Chongqing 400044, China; 20221801038@stu.cqu.edu.cn (L.Y.); sanctuary_97@163.com (X.T.); zhangouyuan1212@163.com (O.Z.); 202418021059@stu.cqu.edu.cn (F.Z.); taocy@cqu.edu.cn (C.T.); 2 Chongqing Sanlei Fiberglass Co., Ltd., Chongqing 409000, China; li_sanlei@163.com; 3 State Key Laboratory of Coal Mine Disaster Dynamics and Control, Chongqing University, Chongqing 400044, China

**Keywords:** aluminosilicate glass, mixed alkaline earth effect, network structure, structure–property relationship, thermodynamics

## Abstract

This work systematically altered the molar ratio of CaO and MgO (R = [CaO]/[(CaO + MgO)], mol%) to elucidate the underlying mechanisms driving the observed changes in macroscopic properties. The results indicated that as CaO increasingly replaced MgO, the rise in the content of non-bridging oxygen led to the depolymerization of the glass structure. A quantitative analysis of Q^n^ units in the [SiO_4_] tetrahedron using ^29^Si MAS NMR revealed that a non-monotonic variation appeared when the Q^4^ unit reached a minimum at R = 0.7. Meanwhile, the chemical environment of aluminum also varies with the R, and the presence of high-coordinated aluminum species is observed when Ca^2+^ and Mg^2+^ ions coexist. In terms of overall performance, both density and molar volume exhibited a linear trend. However, thermal stability, viscosity, characteristic temperatures (including melting temperature, Littleton softening temperature, working point temperature, and glass transition temperature), and mechanical properties showed deviations from linearity. Additionally, four non-isothermal thermodynamics was employed to quantitatively assess the thermal stability of samples C-0.7 and C-1. The insights gained from this study will aid in the development of advanced glass materials with tailored properties for industrial applications.

## 1. Introduction

Aluminosilicate glasses have garnered widespread attention in fields such as electronics, optics, and semiconductors due to their excellent chemical durability [1,2], high mechanical strength [3,4], thermal stability [5,6], and optical performance [7,8]. However, their high contents of SiO_2_ and Al_2_O_3_ inevitably lead to elevated melting temperatures and viscosities, which result in high energy consumption, uneven melting, and difficulty in shaping during industrial production [9,10]. Therefore, how to enhance the processability of aluminosilicate glasses while retaining their desirable properties has become a key challenge in both research and industrial applications [11]. A common strategy to address this problem is to introduce alkaline earth oxides (e.g., CaO, MgO) as network modifiers, which can lower the viscosity and adjust thermal properties [12,13,14,15]. Notably, the co-addition of two alkaline earth oxides can induce non-linear variations in certain glass properties, known as the mixed alkaline earth effect [16,17,18]. This phenomenon has been found to strongly influence dynamic properties like viscosity and glass transition temperature, while static properties such as density and molar volume tend to exhibit more linear behavior [19,20]. This contrast suggests that the mixed alkaline earth effect arises from complex structural interactions, but the fundamental structural origins remain incompletely understood [21,22].

In recent years, the investigation on aluminosilicate glass containing mixed alkaline earth elements has become increasingly prominent. Kjeldsen et al. [23] found that the change the ratio of calcium to magnesium in aluminosilicate glasses affected the local environment around network modifiers. This situation could lead to changes in the glass transition temperature and the liquid fragility of the system, as well as corresponding changes in mechanical properties such as modulus and hardness because of the translational movement of structural units. Shan et al. [15] studied the effects of the mixed alkaline earth effect on structure and properties during the substitution of CaO with MgO and confirmed its structural origin through high-temperature viscosity data fitting. Liu et al. [9] found that during the mutual substitution of CaO and MgO, properties such as glass density, molar volume, and chemical stability exhibited linear changes, while thermal stability and mechanical properties showed deviations from linearity. In terms of the structural origin of the mixed alkaline earth effect, Li et al. [6] proposed that the occurrence of the mixed alkaline earth effect was related to entropy increase, a loose network, and weak bonds rather than network connectivity, based on changes in viscosity, DSC tests, and thermal expansion during the substitution of CaO and MgO. However, most studies lack a comprehensive structural–thermal–property correlation analysis. In addition, the practical value of the mixed alkaline earth effect in tuning glass viscosity and working temperatures for large-scale production has not been sufficiently addressed [24].

In this work, we aim to systematically investigate the structural and thermodynamic consequences of substituting CaO for MgO in aluminosilicate glasses, with emphasis on understanding the origin and application potential of the mixed alkaline earth effect. A series of glasses with varied CaO/(CaO + MgO) ratios were prepared and analyzed via FTIR, ^29^Si and ^27^Al MAS NMR spectroscopy. Thermodynamic behavior was further assessed using DSC and multi-model kinetic analysis. This integrated approach is designed to quantify network depolymerization and changes in Al coordination, evaluate the non-linear thermal response arising from the mixed alkaline-earth effect, and correlate these structural and kinetic changes with industrially relevant processing indicators such as viscosity and thermal workability.

## 2. Experimental Procedure

### 2.1. Glass Sample Preparation

The chemical (XRF, ARL Perform’X, Thermo Fisher Scientific, Ecublens, Switzerland) compositions of the glasses are listed in Table 1. All chemical reagents used in this study were analytical grade. The raw materials were accurately weighed according to predetermined ratios and thoroughly mixed in an agate mortar before being transferred to a 250 mL corundum crucible. The crucible was then placed in a muffle furnace and the temperature was gradually increased to 1853 K at a rate of 10 K/min and maintained at this temperature for 5 h to achieve a homogeneous and bubble-free melt. Part of the molten glass was then quickly poured into a preheated graphite mold, and after forming, transferred to a muffle furnace for annealing at 973 K for 2 h to relieve internal stresses. The remaining molten glass was rapidly quenched in water. After cooling, the glass fragments were ground into powder for future use. The samples were denoted as C-x (x = 0, 0.2, 0.4, 0.5, 0.6, 0.7, 0.8, 1) for simplicity.

### 2.2. Structural Characterization

X-ray diffractometry analyses (PANalytical X’Pert Powder, Almelo, The Netherlands) of the glass samples were conducted in theta-2-theta from 10° to 70° to determine the amorphous nature of the obtained glasses. The overall network structures of the glass samples were recorded using FTIR (FTIR, Nicolet iS50, Thermo Fisher Scientific, Waltham, MA, USA) at the range of 400–1400 cm^−1^, primarily focusing on the variations in [SiO_4_] units within the glass samples [25]. The ^27^Al MAS NMR spectra of glass samples were tested using solid-state NMR spectroscopy (Bruker AVANCE III WB 400 MHz, Ettlingen, Germany) at a Larmor frequency of 104.25 MHz (9.4 T), and the spinning rate was 12 kHz. One-pulse acquisition was used while the cycle delay time was 0.5 s, and the cumulative times were 600. The ^29^Si MAS NMR date was obtained by a (Bruker AVANCE III WB 600 MHz, Ettlingen, Germany) spectrometer at a Larmor frequency of 79.49 MHz. Spinning speeds of 10 kHz at magic angle and 4 mm rotos was chosen. The pulse sequence for ^29^Si MAS NMR data collection was single pulse, with the relaxation delays of 5 s.

### 2.3. Differential Scanning Calorimetry (DSC) Measurement

Using the standard calorimetric method, under argon conditions, a high-temperature differential scanning calorimeter (DSC 404 F3, Netzsch, Selb, Germany) was employed to investigate the impact of the mixed alkaline earth effect on the glass transition temperature (*T_g_*) and thermal stability of the samples during the heating process. The experiments were conducted in an alumina crucible, with a heating rate of 10 K/min for measuring the glass transition temperature. For assessing the thermal stability of the samples, the heating rates were set at 3, 5, 10, 15, and 20 K/min.

### 2.4. Temperature Dependence of Viscosity

By remelting 60 g of crushed glass samples and testing under atmospheric conditions using a rotational cylinder viscometer (RSV 1600, Orton, Westerville, OH, USA), we obtained the variation in glasses viscosity with temperature. Prior to testing, the viscometer was calibrated using standard glass (NIST SRM 717A) to ensure that the logarithmic unit error of the test results was less than 5%. To maintain equilibrium, the melt was held at a set temperature of 1773 K for 30 min. Subsequently, a cooling program was set, and the data were recorded, with the cooling rate set at 2 K/min.

### 2.5. Vickers Microhardness and Bending Strength

The Vickers microhardness of the glass samples was measured using a micro Vickers hardness tester (TUKON 2500, Wolpert Wilson, Norwood, MA, USA) under the conditions of a 0.3 kgf load and a loading time of 10 s. Each glass sample was tested 10 times in parallel to ensure the accuracy and reliability of the results. The bending strength of the glass samples was measured using an electronic–mechanical instrument (Instron 5969, Instron Corporation, Boston, MA, USA). The bulk glass samples had dimensions of 10 × 10 × 4 cm, with a loading speed of 1 mm/min.

### 2.6. Density and Molar Volume

The density of the glass was measured using the Archimedes method [26], with an error range of ±0.005 g/cm^3^, as described in Equation (1):

(1) $$\rho =\frac{m_{1}\cdot \rho_{0}}{m_{1}-m_{2}}$$

where *m*_1_ and *m*_2_ represent the mass of the glass in air and in distilled water, respectively, g, and *ρ*_0_ is the density of distilled water, which is taken as 1 g/cm^3^ for this study. To ensure measurement reliability, each set of glass samples was measured 5 times, and the average value was used. Based on the density of the samples, the molar volume (*V_m_*) [27] of the glass samples was calculated using Equation (2):

(2) $$V_{m}=\sum X_{i}M_{i}∕\rho$$

where *X_i_* represents the mole fraction of each oxide in the composition of glass, *M_i_* denotes the molar mass of each corresponding oxide, and *ρ* is the measured density.

## 3. Results and Discussion

### 3.1. Structural Analysis

#### 3.1.1. XRD Analysis

**Figure 1:**
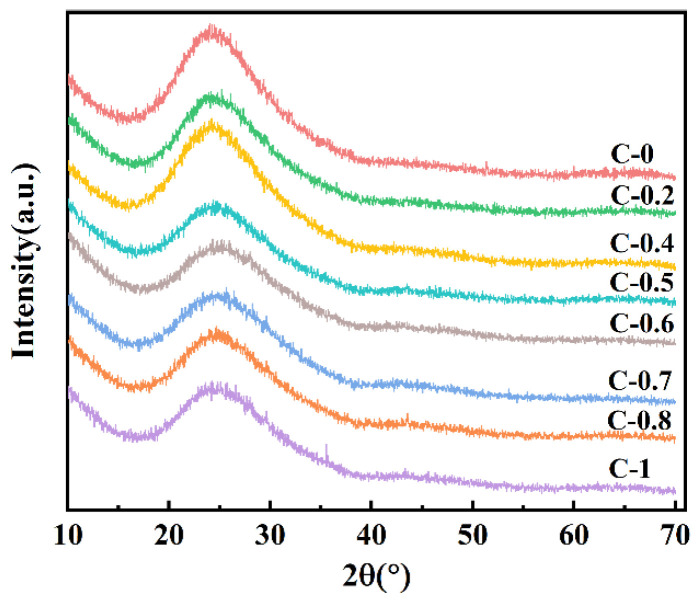
X-ray diffraction patterns of glasses C-0~C-1.

The X-ray diffraction (XRD) patterns of various glass samples are shown in Figure 1. The XRD spectra of the obtained glass samples do not exhibit any distinct peaks corresponding to crystalline phases, confirming the amorphous nature of the samples.

#### 3.1.2. FTIR Spectra Analysis

**Figure 2:**
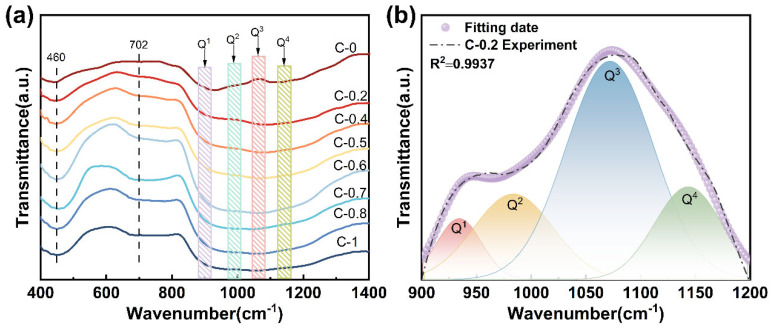
(**a**) FTIR spectra of glasses C-0~C-1; (**b**) deconvolution of FTIR spectrum of glass C-0.2 at 900–1200 cm^−1^.

Figure 2a shows the FTIR spectra of glasses C-0 to C-1 in the range of 400–1400 cm^−1^. Several distinct absorption bands are observed at approximately 460, 702, and 1080 cm^−1^. The band near 460 cm^−1^ is attributed to the bending vibrations of Si-O-Si linkages within [SiO_4_] [28]. The absorption around 702 cm^−1^ originates from the symmetric stretching vibrations of Si-O and Al-O bonds [28]. Notably, the most prominent feature appears near 1080 cm^−1^, corresponding to the asymmetric stretching vibration of Si-O-T bonds (where T = Si or Al) in the [SiO_4_] [9]. To elucidate the effect of mixed alkaline earth modification on the short-range structure of the glass, Gaussian deconvolution was applied to the high-frequency region (900–1200 cm^−1^). Take glass sample C-0.2 as an example: Gaussian fitting produced four fitted curves centered near 950, 1020, 1080, and 1150 cm^−1^, corresponding to Q^1^, Q^2^, Q^3^, and Q^4^ units, respectively, shown as Figure 2b. In Q^n^ groups, *n* is the number of bridge oxygen (BO) connected with [SiO_4_] [29].

The detailed fitting results are summarized in Table 2. The relative area of Q^n^ units exhibit a non-linear evolution with an increasing R value, indicating the presence of a mixed alkaline earth effect. Specifically, at R = 0.7, the Q^2^ units reach their maximum content, accompanied by a corresponding decrease in Q^3^ and Q^4^ units, suggesting enhanced network depolymerization. This phenomenon is primarily attributed to the weaker field strength and larger ionic radius of Ca^2+^ compared to Mg^2+^, which promotes the formation of non-bridging oxygens (NBOs) [15], thereby loosening the glass network. However, when R increases beyond 0.7, the content of Q^2^ units decreases while that of Q^3^ and Q^4^ units increases. This inversion may result from the mismatch in ionic radius and charge density between Ca^2+^ and Mg^2+^, which disrupts homogeneous charge compensation and induces structural rearrangement [9,16,24]. Consequently, the connectivity of the glass network undergoes a non-linear transformation due to the competing structural roles of the two modifying cations.

#### 3.1.3. NMR Analysis

**Figure 3:**
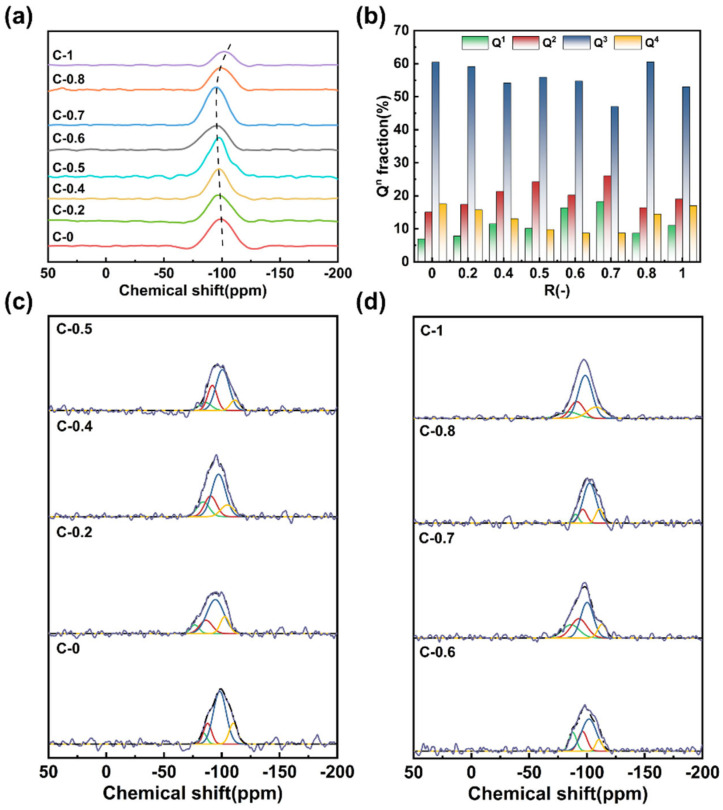
(**a**) ^29^Si MAS NMR spectra of glasses C-0~C-1; (**b**) Q^n^ distribution of [SiO_4_] tetrahedron in the glasses; (**c**,**d**) the Gaussian fitting results of the glasses (R^2^ > 0.95).

In order to further elucidate the network structure connectivity in the glass, ^29^Si MAS NMR spectra and ^27^Al MAS NMR spectra are employed to explain and explore the structural characteristics of the glass. ^29^Si MAS NMR spectra were recorded for all compositions (Figure 3a). Each spectrum exhibits a broad resonance ranging from approximately −70 to −110 ppm, characteristic of tetrahedrally coordinated Si in different Q^n^ (n = 1, 2, 3, 4) environments [27]. With increasing CaO content, the center of mass of the resonance shifts toward lower fields, indicating a decrease in the average number of bridging oxygen atoms and thus a progressive depolymerization of the silicate network [30]. To quantify the distribution of Q^n^ species, the spectra were deconvoluted using a Gaussian fitting approach with peak constraints. The fitting ranges were Q^1^ (−68 to −76 ppm), Q^2^ (−80 to −90 ppm), Q^3^ (−90 to −95 ppm), and Q^4^ (−100 to −110 ppm) [27]. The fitting results are presented in Figure 3c,d, and the corresponding compositional dependence of Q^n^ fractions is shown in Figure 3b. The results indicate that the glass structure is predominantly composed of Q^3^ units (55–65%) and Q^2^ (15–25%). The presence of a significant fraction of Q^3^ species indicates that most modifier cations effectively charge-balance the [AlO_4_]^−^ units. However, the detectable presence of Q^2^ units, especially around R = 0.7, reflects local depolymerization, possibly arising from cation field strength mismatch between Ca^2+^ and Mg^2+^, which may reduce the charge compensation efficiency and promote the formation of non-bridging oxygens (NBOs). These interpretations are also supported by the FTIR results, with which the NMR-derived Q^n^ distributions show strong agreement.

**Figure 4:**
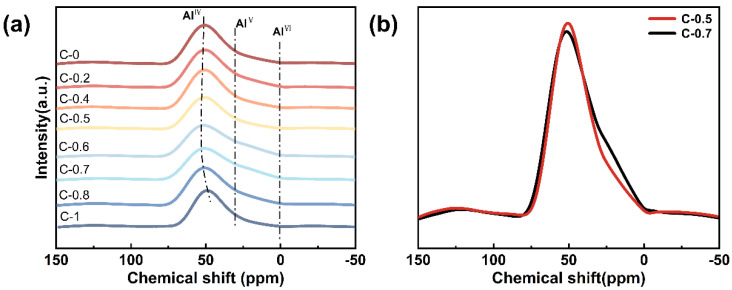
(**a**) ^27^Al MAS NMR spectra of glasses C-0~C-1; (**b**) comparative analysis of ^27^Al MAS NMR spectra for glasses C-0.5 and C-0.7.

The ^27^Al MAS NMR spectra of the glasses are shown in Figure 4a. All spectra exhibit a dominant resonance peak centered around 55 ppm, which is characteristic of tetrahedrally coordinated aluminum (Al^IV^) [31]. This suggests that the majority of Al^3+^ ions are incorporated as network formers in the silicate framework. In addition, minor broadening or shoulders are observed in the range of approximately 0–20 ppm, particularly pronounced in sample C-0.7 (Figure 4b). This feature is commonly associated with the presence of a small fraction of five- or six-coordinated aluminum (Al^V^ or Al^VI^) [32], as reported in prior studies on similar aluminosilicate systems [33]. Specifically, the substitution of Mg^2+^ ions by Ca^2+^ ions influences the local environment of Al^3+^ ions by modifying the field strength and spatial distribution of charge-compensating cations [31]. In contrast, Ca^2+^ has a weaker field strength and tends to induce more structural relaxation and distortion [23]. As evidenced by the increase in Q^2^ units from FTIR and ^29^Si MAS NMR analysis, this also leads to local structural rearrangements around aluminum sites, reflected in the increased heterogeneity of the aluminum coordination environments. Such structural adjustments can reduce network rigidity and symmetry. Overall, the ^27^Al MAS NMR results corroborate the evolving role of mixed alkaline earth modifiers in perturbing the local environment of Al^3+^, thus contributing to the systematic variation in glass properties.

### 3.2. Thermodynamic Behaviors

**Figure 5:**
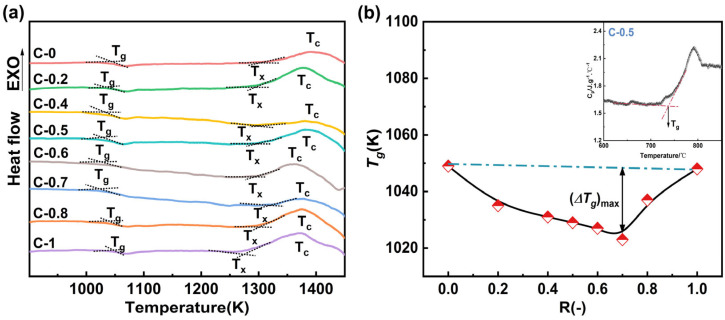
(**a**) DSC curves of glasses C-0~C-1 showing *T_g_* (glass transition), *T_x_* (initial crystallization) and *T_c_* (maximum crystallization); (**b**) composition dependence of *T_g_* as a function of R (inset: illustration of *T_g_* determination).

The DSC analysis curves of the glass samples are shown in Figure 5a. The curves highlight three characteristic temperatures: the glass transition temperature (*T_g_*), the initial crystallization temperature (*T_x_*), and the peak crystallization temperature (*T_c_*). As shown in Figure 5a, all glass samples exhibit a distinct glass transition. Figure 5b shows the variation trend of *T_g_* with the compositional parameter R (*T_g_* values were obtained from the DSC curves of the glass using the method illustrated in the inset of Figure 5b) [34]. The results indicate that the *T_g_* of the glass samples shows non-linear variation with increasing CaO content. According to the topological constraint theory, *T_g_* is inversely proportional to the average degrees of freedom of network atoms at the glass transition [15]. From a thermodynamic perspective, mixed modifiers increase the ionic rearrangement energy barrier compared to single modifiers, which leads to an increase in the configurational entropy of the melt and a decrease in its polymerization degree [35]. The mixed alkaline earth effect reduces the polymerization degree of the melt, which results in an increase in the average degrees of freedom of the atoms in the glass. Consequently, *T_g_* initially decreases and then increases with the addition of CaO.

The stability of the glass, or its resistance to crystallization, was evaluated using the Saad and Poulain method [36] (*K_sp_* = (*T_c_* − *T_x_*)·(*T_x_* − *T_g_*)/*T_g_*). A higher *K_sp_* value indicates better stability. The specific test results are shown in Table 3, the *K_sp_* value exhibits a trend of initially decreasing and then increasing with increasing CaO content. The overall results demonstrate a negative deviation from linearity, with the deviation peaking at R = 0.7. Combining the FTIR spectra results and structural analysis reveals that the substitution of MgO with CaO reduces the stability of the glass network, making atomic diffusion, migration, and rearrangement easier during crystallization [37].

To accurately compare the crystallization resistance of different glasses, we utilized thermodynamic data obtained under various heating rates to calculate the thermodynamic parameters of glasses C-0.7 and C-1 using the isoconversional method. Four non-isothermal kinetic models were employed: Kissinger [38], Ozawa [39], Vyazovkin [40], and Kissinger–Akahira–Sunose (KSA) [41]. By comparing the results calculated from these different models, a better understanding of the thermal stability of the glasses was achieved.

1. Kissinger kinetic models: The thermodynamic parameters of the model are calculated using Equation (3):

(3) $$\mathrm{In}\left(\frac{\beta }{T^{2}}\right)=\;\mathrm{In}\left(\frac{\mathrm{AR}}{E_{a}}\right)-\frac{E_{a}}{\mathrm{RT}}$$

where *A* is the pre-exponential factor; *α* is the conversion degree during the reaction; *β* is the heating rate, K/min; *T* is the reaction temperature, K; and *R* is the universal gas constant, R = 8.314 J/(mol·K).

2. The Ozawa kinetic model: The thermodynamic parameters of the model are calculated using Equation (4):

(4) $$\;\mathrm{In}\beta \;=\;\mathrm{In}\left[\frac{AE_{a}}{\mathrm{RG}\left(\alpha \right)}\right]-\;2.315\;-\;0.4567\frac{E_{a}}{\mathrm{RT}}$$

where *G*(*α*) is the conversion degree integral function. When *α* is fixed, *G*(*α*) is also fixed.

3. The Vyazovkin kinetic model: The thermodynamic parameters of the model are calculated using Equation (5):

(5) $$\;-\mathrm{In}t_{\alpha ,\;t}\;=\;\mathrm{In}\left[\frac{A}{G\left(\alpha \right)}\right]-\;\frac{E_{a}}{\mathrm{RT}}$$

where *t_α, t_* is the time required to reach different conversion rates in the reaction.

4. The KSA kinetic model: The thermodynamic parameters of the model are calculated using Equation (6):

(6) $$\mathrm{In}\left(\frac{\beta }{T^{2}}\right)=\;\mathrm{In}\left[\frac{\mathrm{AR}}{E_{a}g\left(\alpha \right)}\right]-\frac{E_{a}}{\mathrm{RT}}$$

**Figure 6:**
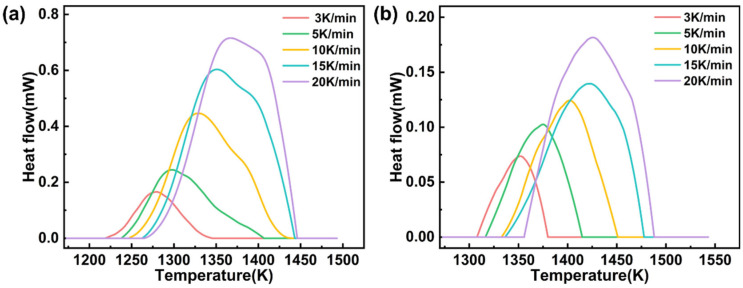
DSC exothermic curves of glasses C-0.7 (**a**) and C-1 (**b**) at various *β* values (3, 5, 10, 15, and 20 K/min).

The heat flow curves of glasses C-0.7 and C-1 under five *β* values (3, 5, 10, 15, and 20 K/min) in the DSC measurement are shown in Figure 6. The Y-axis values of the curve peaks represent the maximum heat flow of the two glasses during the reaction. Moreover, as *β* increases, the heat flow curves of both glasses shift towards higher temperatures, and the peak values correspondingly increase.

**Figure 7:**
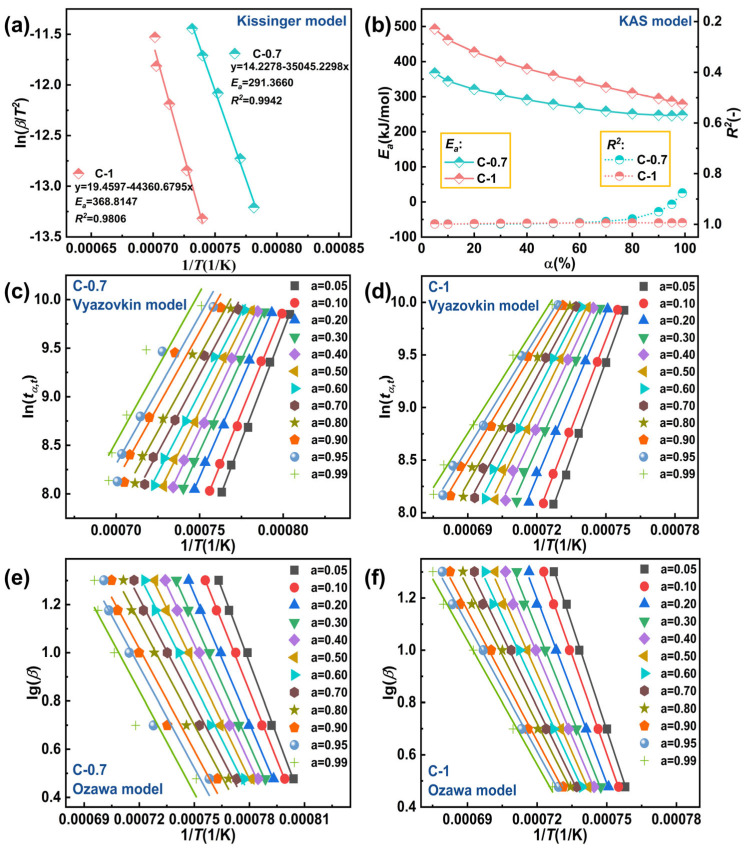
(**a**) *E_a_* values of glasses C-0.7and C-1 at various *β* values with the Kissinger kinetic method; (**b**) plots of calculated *E_a_* values versus *α* of glasses C-0.7 and C-1 with the KAS kinetic model; (**c**) glass C-0.7 with the Vyazovkin kinetic model; (**d**) glass C-1 with the Vyazovkin kinetic model; (**e**) glass C-0.7 with the Ozawa kinetic model; (**f**) glass C-1 with the Ozawa kinetic model.

Additionally, based on the thermodynamic data obtained from DSC, the activation energy (*E_a_*) of glasses C-0.7 and C-1 were calculated using the aforementioned four non-isothermal kinetic models. The kinetic parameters were determined using the Kissinger dynamic model, and the relationship between different β values and temperature is shown in Figure 7a. The *E_a_* values of glasses C-0.7 and C-1 were found to be 291.3660 kJ/mol (*R*^2^ = 0.9942) and 368.8147 kJ/mol (*R*^2^ = 0.9806), respectively. To assess the reliability of the Kissinger dynamic model, the Kissinger–Akahira–Sunose (KAS) dynamic method was developed [42]. Since the selection of baseline during analysis is subjective, for the Ozawa, Vyazovkin, and KSA models, we deliberately chose 12 conversion rates (*α* = 0.05, 0.1, 0.2, 0.3, 0.4, 0.5, 0.6, 0.7, 0.8, 0.9, 0.95, and 0.99) for thermodynamic analysis [29]. Figure 7b illustrates the relationship between *E_a_* and *R*^2^ at different conversion rates (*α*) under the KAS dynamic model. After calculation using this model, the overall *R*^2^ of glasses C-0.7 and C-1 were close to the ideal value of 1.0, with *E_a_* values ranging from approximately 200 to 500 kJ/mol. The Vyazovkin (Figure 7c,d) and Ozawa (Figure 7e,f) dynamic models adopted the same conversion rates as the KAS dynamic model to ensure fair comparison of linear fitting models under different isoconversional conditions. Table 4 and Table 5 provide the calculated *E_a_* values and *R*^2^ of glasses C-0.7 and C-1 using the two kinetic models.

**Figure 8:**
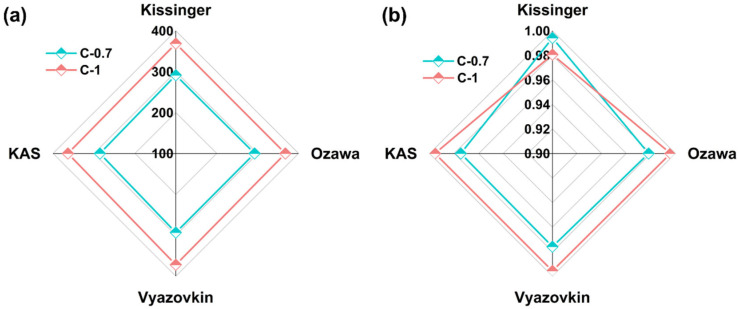
*E_a_* and *R*^2^ of glasses C-0.7 and C-1 at various *β* values with Kissinger, KAS, Ozawa, and Vyazovkin kinetic models. (**a**) *E_a_* of glasses; (**b**) *R^2^* of glasses.

The results of *E_a_* and *R*^2^ for the two glass samples using the four non-isothermal models are shown in Figure 8 and Table 6. It can be observed that the *E_a_* value of glass C-0.7 is 283.2120 kJ/mol (*R*^2^ = 0.9808), while for glass C-1, the *E_a_* value is 368.1595 kJ/mol (*R*^2^ = 0.9920). The *E_a_* value of glass C-1 slightly higher than that of glass C-0.7. A higher *E_a_* value indicates a higher energy barrier to overcome during glass crystallization, implying greater difficulty in crystallization. Therefore, glass C-1 exhibits higher thermal stability compared to glass C-0.7. It should be emphasized that the kinetic analyses conducted herein, though comprehensive in employing multiple heating rates and isoconversional methodologies, were limited to two representative glass compositions (C-0.7 and C-1). While this approach effectively illustrates the influence of compositional extremes on crystallization kinetics, extending such analyses across a broader compositional range would be essential for deriving universally applicable kinetic models. Future investigations will aim to systematically explore this parameter space to deepen the mechanistic understanding of mixed alkaline earth effects on the crystallization resistance of aluminosilicate glasses.

### 3.3. Overall Performance

#### 3.3.1. Density and Molar Volume

**Figure 9:**
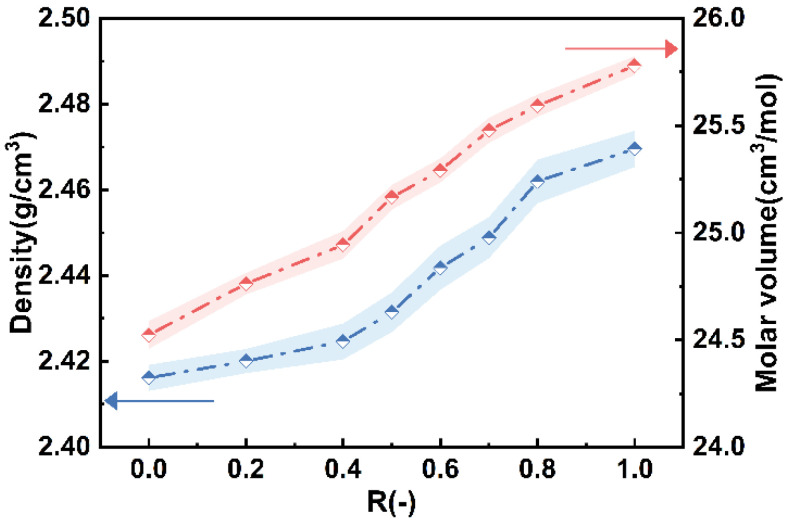
Variations in glass densities and molar volume with R.

As shown in Figure 9, the density and molar volume of the glass samples show a near-linear increase with the increasing CaO content. The density of the glass samples rises from 2.42 g/cm^3^ to 2.47 g/cm^3^, while the molar volume increases from 24.52 cm^3^/mol to 25.78 cm^3^/mol. Typically, the density of glass is determined by the average molar mass of the atoms in its composition [26]. Ca^2+^ has a larger molar mass and ionic radius, which results in higher glass density and molar volume for glasses with higher CaO content.

#### 3.3.2. Rheological Properties

**Figure 10:**
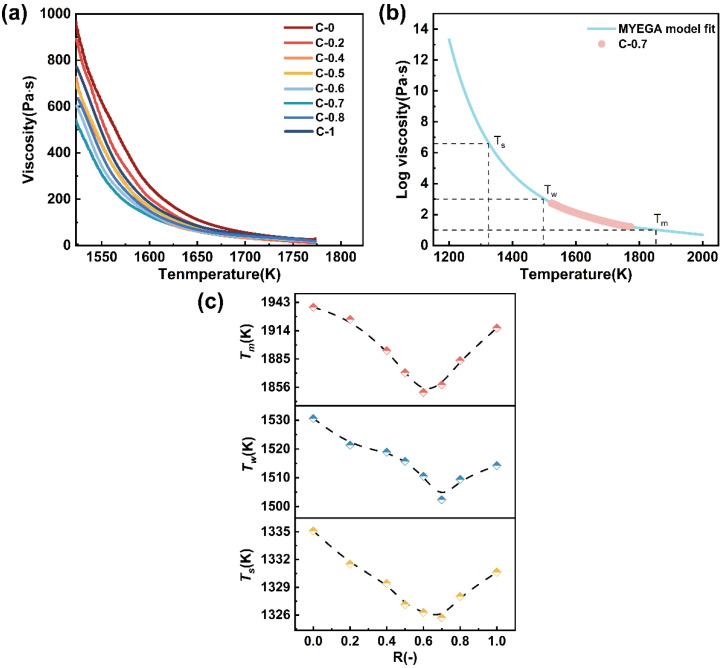
(**a**) The viscosity–temperature curve for glass samples C-0~C-1; (**b**) viscosity as a function of temperature for glass sample 0.7 fitted by the MYEGA equation; (**c**) the composition dependence of *T_m_*, *T_w_*, and *T_s_* as a function of R.

The viscosity of the melt reflects the ability of the glass melt to resist flow, which is closely related to the degree of network structure polymerization and atomic diffusion within the glass [43]. This study measured the high-temperature viscosity of glasses C-0~C-1, with the results shown in Figure 10a. It can be seen that as the temperature increases, the viscosity of the glasses gradually decreases. Additionally, when the temperature exceeds 1700 K, the viscosity of the glasses approaches a minimum value. As CaO continuously replaces MgO, the viscosity of the glasses initially decreases and then increases, indicating the occurrence of the mixed alkaline earth effect due to the coexistence of both elements [44].

The relationship between melt viscosity and temperature is of critical importance for glass manufacturing and subsequent processing. Among the various viscosity–temperature models, the MYEGA model is particularly effective in capturing the changes in configurational entropy and provides a more accurate prediction of the viscosity–temperature curve across the entire temperature range [44]. The model is given by Equation (7):

(7) $$\mathrm{lg}\eta \left(T\right)\;=\;\mathrm{lg}\eta_{\infty }\;+\;\left(12\;-\;\mathrm{lg}\eta_{\infty }\right)\frac{T_{g}}{T}\exp\left[\left(\frac{m}{12-\mathrm{lg}\eta_{\infty }}\;-\;1\right)\left(\frac{T_{g}\;}{T}\;-\;1\right)\right]$$

where lg*η_∞_* is the logarithm of the viscosity at infinite temperature; *T_g_* is the glass transition temperature, K; and *m* is the liquid fragility index. Taking glass sample C-0.7 as a representative, the viscosity–temperature relationship was fitted using the MYEGA model, as shown in Figure 10b. The melting temperature (*T_m_*, *η* = 10^1^ Pa·s), the working point (*T_w_*, *η* = 10^3^ Pa·s), and the Littleton softening temperature (*T_s_*, *η* = 10^6.6^ Pa·s) can be obtained from the fitting results. Figure 10c shows the temperature points corresponding to the characteristic viscosity points as a function of glass composition R.

It can be observed that all three characteristic temperatures exhibit a non-linear compositional dependence, reaching their minimum values around R = 0.6~0.7. This indicates that glasses within this compositional range possess enhanced thermoplasticity and improved processability at elevated temperatures. Typically, this viscosity behavior can be directly correlated with the structural depolymerization induced by varying the alkaline earth modifier [16,45]. A combined analysis of the FTIR and ^29^Si MAS NMR results reveals that in the range of R = 0.6–0.7, the proportion of Q^2^ units increases significantly, while the fractions of Q^3^ and Q^4^ units decrease, indicating the highest degree of network depolymerization within this range. As R increases further, the fractions of Q^3^ and Q^4^ units exhibit a rebound, which may be attributed to local repolymerization induced by high CaO content or the partial participation of Ca^2+^ in the network formation. This leads to enhanced local cross-linking and increased structural compactness, thereby resulting in a viscosity increase [15,27]. The coupled evolution of network structure and viscosity behavior suggests that both the type and proportion of alkaline earth modifiers not only regulate the connectivity of the glass network but also play a critical role in determining its high-temperature rheological properties.

#### 3.3.3. Vickers Hardness Analysis (H_V_) and Bending Strength

**Figure 11:**
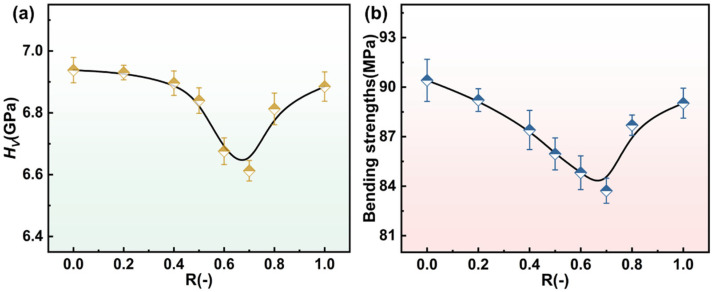
The variation in Vickers hardness (**a**) and bending strength (**b**) of glasses C-0~C-1 with R.

The variation in Vickers hardness and bending strength with the composition parameter R is shown in Figure 11a,b. Both properties exhibit a non-linear trend; they initially decrease and then increase as R increases. This behavior aligns with the structural evolution induced by the mixed alkaline earth effect [16,24]. In the intermediate composition range (R = 0.6–0.7), a decline in mechanical properties is observed, which correlates with the peak in Q^2^ units and increased network depolymerization, as evidenced by FTIR, ^29^Si and ^27^Al MAS NMR spectra. Indeed, the field strength difference between Mg^2+^ (0.46 Å^−2^) and Ca^2+^ (0.36 Å^−2^) plays a crucial role. The substitution of higher-field-strength Mg^2+^ by Ca^2+^ facilitates the formation of non-bridging oxygens (NBOs), leading to a more depolymerized network. As Ca^2+^ content increases further, partial repolymerization likely occurs due to enhanced charge compensation by AlO_4_^−^ tetrahedra and the increasing relative influence of Ca^2+^ in cross-linking the network, which restores some rigidity and thus improves the mechanical properties [46,47]. These results collectively elucidate how the interplay among cation field strength, network depolymerization (as reflected by the Q^2^/Q^3^/Q^4^ unit distributions), and charge compensation mechanisms governs the mechanical response of the glass system.

## 4. Conclusions

This study elucidates the structural and thermodynamic implications of CaO–MgO substitution in aluminosilicate glasses, with a particular emphasis on the manifestation of the mixed alkaline earth effect. Spectroscopic analyses, including FTIR, ^29^Si and ^27^Al MAS NMR, revealed pronounced non-linear variations in Q^n^ species distribution and aluminum coordination, indicating a systematic reorganization of the glass network structure as a function of the CaO/MgO ratio. Kinetic analyses based on multiple models (Kissinger, Ozawa, Vyazovkin, and KAS) performed on two selected glass compositions (C-0.7 and C-1) revealed distinct differences in crystallization activation energy, providing thermodynamic insights into how varying CaO/MgO ratios can influence glass stability. Viscosity–temperature relationships modeled via the MYEGA equation showed that the characteristic temperatures (*T_m_*, *T_w_*, and *T_s_*), each a minimum, at intermediate CaO/MgO ratios (R = 0.7), suggesting improved thermal processability. Meanwhile, the Vickers hardness and bending strength exhibit non-linear variations, which can be attributed to a reduction in network compactness induced by the synergistic interaction between Ca^2+^ and Mg^2+^ modifiers. These findings offer practical guidance for designing aluminosilicate glasses with optimized processability and controlled thermal properties. The identified composition range around R = 0.7, exhibiting enhanced depolymerization and reduced characteristic temperatures, may be particularly advantageous for applications such as insulation glass fibers and sealing materials, where balanced viscosity, thermal stability, mechanical properties, and controlled crystallization behavior are critical.

## Figures and Tables

**Table 1:** The chemical composition of the glass samples in this work (mol%).

Glass ID	SiO_2_	Al_2_O_3_	MgO	CaO	[CaO]/[(CaO + MgO)] = R
C-0	59.48	19.81	20.27	0.44	0
C-0.2	59.11	19.94	16.28	4.67	0.2
C-0.4	59.47	20.02	12.36	8.15	0.4
C-0.5	59.58	19.87	10.32	10.23	0.5
C-0.6	59.76	19.91	8.42	11.90	0.6
C-0.7	59.73	19.87	6.47	13.93	0.7
C-0.8	59.87	20.01	4.45	15.66	0.8
C-1	59.03	20.05	1.06	19.87	1

**Table 2:** Frequencies (*V_i_*) and relative area (*A_i_*) of FTIR spectra of glasses C-0~C-1 obtained by peak fitting.

Glass ID	C-0	C-0.2	C-0.4	C-0.5	C-0.6	C-0.7	C-0.8	C-1
*V*_1_/cm^−1^	932	934	937	939	943	946	948	949
*V*_2_/cm^−1^	980	983	994	1000	1003	1006	1011	1014
*V*_3_/cm^−1^	1070	1071	1077	1080	1083	1087	1093	1098
*V*_4_/cm^−1^	1140	1143	1145	1148	1150	1153	1154	1154
*A*_1_/%	4.36	7.56	9.66	11.47	12.88	14.04	11.21	8.57
*A*_2_/%	15.24	19.49	21.83	23.93	24.95	26.04	22.36	19.35
*A*_3_/%	60.81	55.49	53.95	51.23	50.57	49.51	52.87	54.84
*A*_4_/%	19.59	17.46	14.56	13.37	11.60	10.41	13.56	17.24

**Table 3:** The thermal physical properties of glasses C-0~C-1.

Glass ID	C-0	C-0.2	C-0.4	C-0.5	C-0.6	C-0.7	C-0.8	C-1
*T_c_*/K	1398	1380	1380	1375	1360	1375	1378	1372
*T_x_*/K	1306	1291	1295	1300	1285	1320	1301	1277
*T_g_*/K	1049	1035	1031	1029	1027	1023	1037	1045
*K_sp_*/K	22.54	22.01	21.77	19.75	18.84	15.97	19.60	20.76

**Table 4:** *E_a_* and *R*^2^ of glass C-0.7 using three models (Ozawa, Vyazovkin, an KAS) with various values of *α*.

*α*	Ozawa		Vyazovkin		KAS	
	*E_a_* (kJ/mol)	*R* ^2^	*E_a_* (kJ/mol)	*R* ^2^	*E_a_* (kJ/mol)	*R* ^2^
0.05	369.6421	0.9984	374.8650	0.9983	367.4868	0.9983
0.10	347.7730	0.9989	351.7931	0.9989	344.3253	0.9988
0.20	325.4120	0.9990	328.1821	0.9989	320.5989	0.9988
0.30	310.4874	0.9986	312.4150	0.9985	304.7454	0.9984
0.40	297.8705	0.9981	299.0807	0.9979	291.3322	0.9978
0.50	286.1455	0.9968	286.6881	0.9965	278.8653	0.9963
0.60	275.9938	0.9941	275.9485	0.9935	268.0499	0.9932
0.70	267.1689	0.9902	266.5996	0.9892	258.6202	0.9886
0.80	260.2941	0.9816	259.2983	0.9796	251.2344	0.9784
0.90	256.8398	0.9574	255.5797	0.9528	247.4150	0.9500
0.95	255.5037	0.9322	254.1167	0.9250	245.8842	0.9205
0.99	257.5316	0.8938	256.1650	0.8829	247.8344	0.8765
Average	292.5552	0.9783	293.3943	0.9760	285.5327	0.9746

**Table 5:** *E_a_* and *R*^2^ of glass C-1 with various *α* values.

*α*	Ozawa		Vyazovkin		KAS	
	*E_a_* (kJ/mol)	*R* ^2^	*E_a_* (kJ/mol)	*R* ^2^	*E_a_* (kJ/mol)	*R* ^2^
0.05	489.9266	0.9988	500.8239	0.9987	492.8134	0.9986
0.10	460.7073	0.9994	470.0441	0.9993	461.9714	0.9993
0.20	427.9000	0.9975	435.4660	0.9973	427.3017	0.9972
0.30	403.6348	0.9964	409.8850	0.9961	401.6461	0.9960
0.40	382.7767	0.9962	387.8940	0.9959	379.5888	0.9960
0.50	364.6916	0.9957	368.8233	0.9954	360.4570	0.9952
0.60	349.0043	0.9953	352.2743	0.9949	343.8471	0.9947
0.70	332.8259	0.9952	335.2083	0.9948	326.7199	0.9945
0.80	317.3123	0.9947	318.8352	0.9942	310.2784	0.9939
0.90	302.8900	0.9949	303.6022	0.9944	294.9686	0.9941
0.95	295.3999	0.9950	295.6843	0.9946	287.0030	0.9943
0.99	287.5848	0.9944	287.4148	0.938	278.6746	0.9934
Average	367.8879	0.9961	372.1692	0.9958	363.7725	0.9956

**Table 6:** The average *E_a_* and *R*^2^ of glasses C-0.7 and C-1 at various *β* values with the Kissinger, KAS, Ozawa, and Vyazovkin kinetic models.

	C-0.7			C-1		
	*E_a_* (kJ/mol)	*R* ^2^		*E_a_* (kJ/mol)	*R* ^2^	
Kissinger	291.3660	0.9942		368.8147	0.9806	
Ozawa	292.5552	0.9783		367.8879	0.9961	
Vyazovkin	293.3943	0.9760		372.1629	0.9958	
KAS	285.5327	0.9746		363.7725	0.9956	
Aaverage	283.2120	0.9808		368.1595	0.9920	

## Data Availability

The original contributions presented in this study are included in the article. Further inquiries can be directed to the corresponding author.
